# Functional super-resolution microscopy of fibers and polymers: convergence of artificial and biological systems at the nanoscale

**DOI:** 10.1039/d5nh00729a

**Published:** 2025-12-17

**Authors:** Si-Jia Rao, Xiayi Gong, Md Abul Shahid, Yunshu Liu, Hongjing Mao, Yang Zhang

**Affiliations:** a Molecular Analytics and Photonics (MAP) Lab, North Carolina State University Raleigh North Carolina 27606 USA yang.zhang@ncsu.edu; b Fiber and Polymer Science Program, Department of Textile Engineering, Chemistry and Science, North Carolina State University Raleigh North Carolina 27606 USA; c Lampe Joint Department of Biomedical Engineering, University of North Carolina at Chapel Hill and North Carolina State University Chapel Hill NC 27599 USA

## Abstract

Fluorescence nanoscopy has opened a new frontier for visualizing and understanding polymeric and fibrous materials with molecular precision. Building on advances in single molecule localization microscopy (SMLM), researchers are now extending beyond structure to probe dynamic and functional properties that govern material behavior. This Focus article highlights recent progress in functional SMLM for mapping polarity, viscosity and molecular motion within polymers and fibers, revealing how these nanoscale parameters influence macroscopic performance. Examples include tracking polymerization and phase evolution, resolving nanofiber organization, and correlating structural heterogeneity with local chemical environments. We further discuss the growing convergence between artificial and biological systems with shared principles of hierarchical organization. By integrating structural, dynamic, and functional imaging, fluorescence nanoscopy provides a unifying framework for studying and engineering complex molecular assemblies across living and synthetic matter.

## Abstract 2

The diagram highlights functional SRM as a bridge between nanoscale studies of biological and artificial fibers and polymers where advances in SRM enable study of synthetic systems and polymer principles guide understanding of complex natural fiber.

## Graphical abstract

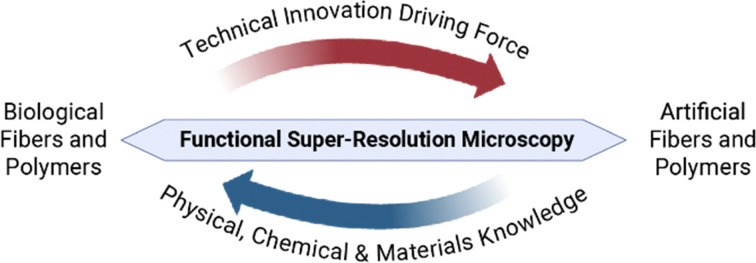

## Introduction

**Fig. 1:**
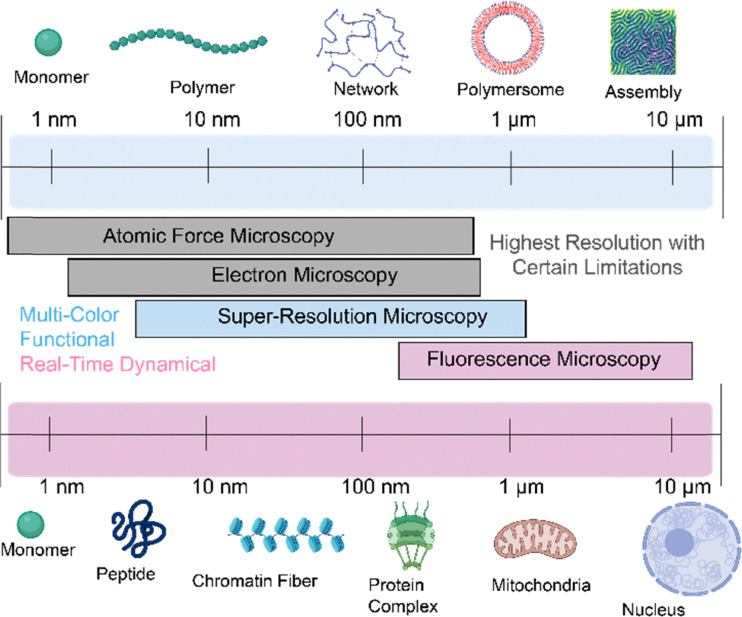
Length scale from monomers to microscopic fibres and assemblies in synthetic and biological systems along with the imaging resolution of existing nanoscopic imaging technologies.

Polymeric materials constitute some of the most versatile and dynamic classes of functional matter, owing to their structural diversity, chemical tunability, and scalable processability. The fundamental concept of fibers and polymers as macromolecules composed of repeating units enables precise molecular design and hierarchical assembly from the molecular to the nanoscale (Fig. 1 top), thereby imparting tailored physicochemical properties and multi-functionality.^1–4^ While conventional applications span food packaging,^5,6^ coatings,^7,8^ and textiles,^9^ the past decades have witnessed the emergence of polymer and fiber systems as indispensable components in advanced technologies, including flexible electronics,^10–12^ nano medicine,^13,14^ energy storage and conversion,^15,16^ and sustainable materials design.^17,18^ Their inherent capacity for self-assembly, responsiveness to external stimuli, and compatibility with hybrid nanostructures uniquely position polymers at the interface of fundamental nanoscience and translation innovation. A comprehensive understanding of the structure and function of polymeric materials at the nanoscale is essential for advancing the design of next-generation materials across diverse applications.^19^ This requirement becomes even more pronounced for complex molecular systems, where their nanoscale structural, functional and dynamic behaviors present challenges for characterization using conventional nanoscopic imaging approaches such as atomic force microscopy (AFM) and electron microscopy (EM).^20^ EM primarily captures static structural snapshots under non-native or vacuum conditions, lacking the spatiotemporal resolution and environmental compatibility needed to visualize the dynamic and functional processes of polymers at the single-molecule or nanoscopic level (Fig. 1 middle). AFM can operate in liquid and under physiological conditions, allowing the observation of dynamic biological processes and enabling mechanical measurements, viscosity studies, and other biophysical analyses. For instance, AFM has been used to capture the motion of bacteriorhodopsin, myosin on actin, and the DNA double helix at high resolution.^21,22^ In turn, AFM also has inherent limitations in imaging depth which is mostly restricted to surfaces, making it difficult to probe structures within thicker samples.

Fluorescence microscopy stands out for its inherent advantages, including real-time visualization, imaging depth of tens of micrometers and non-invasive observation. With the advent of novel fluorescent probes,^23,24^ the development of diverse optical strategies,^25,26^ fluorescence super-resolution microscopy (SRM)^27–29^, emerged in the early 21st century as a groundbreaking advancement that surpassed the optical diffraction limit of improving the spatial resolution of optical microscopy from ∼200 nm down to a few nanometers. SRM has rapidly evolved into an indispensable tool in life science research, offering unprecedented capabilities for probing subcellular fibers (*e.g.*, cytoskeletons and chromatin fibers), assemblages (*e.g.*, plasma and organelle membranes), and individual biomacromolecules (*e.g.*, proteins and nucleic acids) as well as their dynamics at the nanoscale (Fig. 1 bottom).^30–33^ Benefiting from these breakthroughs, the invention of SRM was awarded the 2014 Nobel Prize in Chemistry.^25,34,35^ A wide body of scholarly reviews has emerged, each highlighting different facets of SRM. These reviews include critical discussions on innovations in imaging methodologies,^36–40^ the development of switchable and functional fluorophores,^24,39,41–44^ the integration of advanced spectroscopic approaches,^45^ and the expanding landscape of applications in the life sciences.^46–48^ Together, these perspectives have established a rich foundation that continues to guide both technological innovation and interdisciplinary exploration.

The convergence of physical and life sciences at the polymer nanostructure level has naturally extended this progress to materials research, as biomacromolecules assemble the molecular backbone of biological systems and share fundamental structural principles with synthetic macromolecules. This conceptual continuity makes it both intuitive and powerful to adapt SRM from visualizing biological polymers to investigating synthetic polymeric materials, enabling direct observation of molecular organization, phase transitions, and dynamic processes that define their macroscopic properties.

In this Focus article, we introduce the principles of emerging SRM methods for visualizing dynamic and functional properties of polymeric and fibrous materials. Prior reviews^49–54^ have traced SRM's evolution from a biological imaging tool to a core analytical technique in materials characterization, highlighting its applications in supramolecular polymers,^49^ dynamic self-assembly, nanocomposites, and bioinspired architectures, as well as its capacity to resolve structure–function relationships under *in situ* conditions.^51^ These studies have established the foundation for applying SRM to polymer systems, emphasizing its growing potential for probing nanoscale organization, phase behaviour, and 3D dynamics. Our Focus article specifically centres on advanced and functional single-molecule localization microscopy (SMLM) as a rapidly developing class of SRM that achieves <10 nm spatial resolution and single-molecule sensitivity. We outline the core concepts and representative modalities of SMLM, assess switchable fluorophores suited for polymer imaging, and highlight recent advances demonstrating how SMLM can reveal molecular-level functional properties of polymers and fibers. We conclude by summarizing current progress, identifying key challenges that remain, and offering our perspective on how studies of polymeric and fibrous materials using SRM can bridge the understanding of artificial and biological systems.

## Overview of conventional SMLM methods

**Fig. 2:**
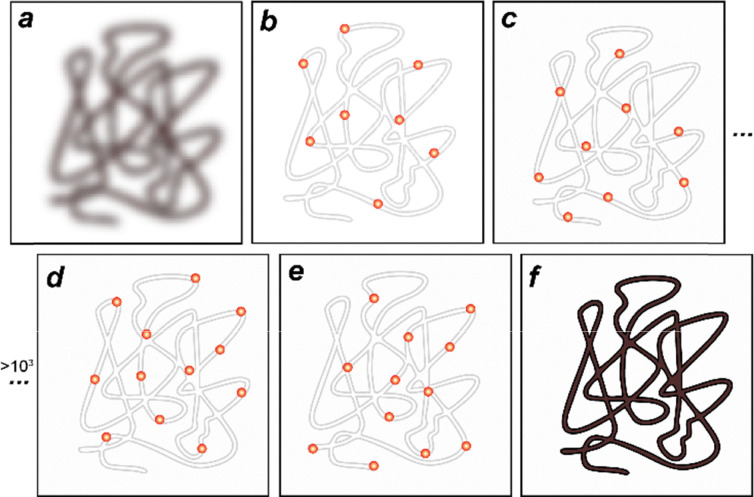
Schematic illustration of SMLM principles. A diffraction-limited blurry fluorescence image of a fiber (a) is separated into sparsely distributed subsets at different times (b)–(e) utilizing the stochastic single-molecule “blinking” process of switchable fluorophores to reconstruct a super-resolved image (f).

SRM can be broadly categorized based on their underlying working principles. The first class includes methods that rely on engineered illumination patterns, such as stimulated emission depletion microscopy (STED)^55^ and structured illumination microscopy (SIM).^56,57^ The second class relies on single-molecule detection with a common name of SMLM including its variants such as stochastic optical reconstruction microscopy (STORM)^28^ and photoactivated localization microscopy (PALM),^22^ as well as point accumulation for imaging in nanoscale topography (PAINT)^58^ and DNA-PAINT.^59^ In terms of spatial resolution, SMLM typically achieves 10–40 nm, STED reaches approximately 50–80 nm, and SIM provides around 100–120 nm lateral resolutions. STED and SIM also support faster imaging rates with approximately 10–50 frame per second (fps) for STED and 1–20 fps for SIM making them well suited for live-cell applications. SMLM can be implemented in live cells, particularly through PALM and PAINT strategies that use cell-permeable, photoswitchable probes, although typical imaging speed (>10 seconds per video for reconstruction) are slower. In contrast, STORM and DNA-PAINT are largely restricted to fixed samples due to specialized buffer requirements and slower acquisition rates. The basic principle of SMLM is described as the following:

In a conventional fluorescence image (Fig. 2a), a single fluorophore appears as a diffraction-limited spot described by the point spread function (PSF), often modeled as a two-dimensional Gaussian with a width of ∼*λ*/2, with *λ* being the emission wavelength of the emitter. Visible light spectral range therefore correspond to PSF sizes of ∼200–300 nm. Because tens and hundreds of fluorophores are typically present within each diffraction-limited region, their emission patterns overlap, making it impossible to resolve individual molecules. To overcome this, the fundamental working principle of SMLM briefly involves iterative imaging of sparsely activated fluorophores across numerous acquisition cycles (Fig. 2b–e). In each frame, the PSF of individual, well-separated single-molecule emitters are captured and subsequently analyzed using localization algorithms to determine their precise spatial coordinates with nanometer precision. with localization precision (*σ*_*xy*_) scaling as 1/√*N*, where *N* is the single-molecule photon count. Pixel size and background noise further influence this accuracy.^60^ By accumulating and reconstructing the localization from all frames, a super-resolved image (Fig. 2f) is generated with nanometer-scale resolution.^61,62^

Assessing the quality or spatial resolution of SMLM images, *σ*_*xy*_ is a key metric. It is commonly estimated from the photon count detected within the fluorescence spot of individual fluorophores.^60^ Alternatively, a practical and experimentally accessible method involves analyzing the nearest-neighbor distances of fluorophores across consecutive frames.^63^ The spatial resolution of a SMLM image can be reported as 2.36 × *σ*_*xy*_ corresponding to the full width at half maximum that defines the minimum separation between two neighbouring emitters that can be resolved. Besides *σ*_*xy*_, labeling density also has to be considered to fulfill the Nyquist sampling theorem.^64^ As an objective measure, Fourier ring correlation (FRC) resolution^65^ quantifies the overall structural reproducibility of the reconstructed image and captures not only *σ*_*xy*_ but also labelling density, and image reconstruction parameters.

A variety of optical strategies have been developed to enable 3D localization of single fluorophores. One of the earliest methods introduced a cylindrical lens into the detection path,^66^ inducing an asymmetric distortion of the PSF that encodes the axial position of the emitter an approach that has since been widely adopted in 3D SMLM.^67^ Alternative techniques include multi-plane detection,^68,69^ and engineered PSFs such as the double-helix PSF,^70^ interferometric setups,^71^ and dual-objective configurations.^72,73^ These methods vary in terms of experimental complexity, attainable localization precision, and the axial imaging range achievable within a single acquisition.^74^ Notably, the axial localization precision *σ*_*z*_ is usually worse than *σ*_*xy*_ which results in anisotropic 3D resolution. FRC can also be used to measure the axial resolution.

### SMLM with photoswitchable fluorophores

**Fig. 3:**
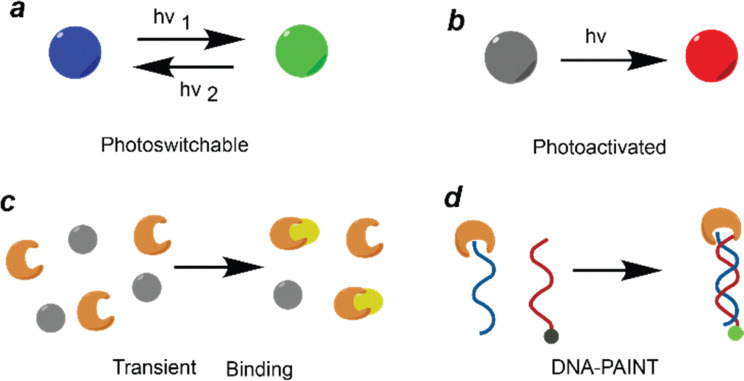
The illustration of SMLM based on (a) reversible photoswitching, (b) irreversible photoactivation, (c) transient binding, and (d) DNA-PAINT (transient hybridization between a dye-conjugated imager and a substrate-labelled docking strand).

The earliest implementations of SMLM (PALM and STORM) use light to control fluorescence switching, temporally isolating the emission of sparse fluorophores and localizing them with nanometer precision to reconstruct super-resolved images. Although PALM and STORM share this fundamental principle, they differ in fluorophore types and photophysical mechanisms. STORM uses synthetic photoswitchable dyes that reversibly transition between dark and bright states (Fig. 3a), offering higher photon yields and superior localization precision due to their brightness. However, STORM requires specialized imaging buffers and is less suited for live-cell imaging. PALM, in contrast, typically employs genetically encoded photoactivatable fluorescent proteins, enabling live-cell imaging through irreversible photoactivation/conversion, single-molecule detection, and irreversible photobleaching (Fig. 3b). Both methods routinely achieve lateral resolutions of ∼20 nm and have become foundational tools for nanoscale biological and materials imaging.

### SMLM based on the transient binding mechanism

PAINT and DNA-PAINT represent complementary approaches within the SMLM family that exploit the transient binding of fluorescent probes. Traditional PAINT^58^ employs freely diffusing fluorophores that transiently and non-specifically associate with sample surfaces, enabling super-resolution imaging without covalent labelling or genetic tagging. However, the stochastic nature of these interactions limits molecular specificity and multiplexing capability. DNA-PAINT, in contrast, leverages sequence-specific hybridization between short “imager” and “docking” DNA strands, allowing programmable and reversible probe binding with high specificity and tunable labeling density.^75^ This molecular programmability provides enhanced spatial resolution (often <10 nm), precise quantification, and facile multiplexed imaging through the exchange of orthogonal imager strands. While both methods reconstruct images by accumulating transient binding events, DNA-PAINT offers superior specificity and versatility, making it particularly suitable for complex biological samples that require multiplexed and quantitative nanoscale imaging, though its slow binding kinetics limit live-cell applications. More recently, coupling environment-sensitive dyes with multi-dimensional SMLM/PAINT^26,76,77^ setups has extended the technique beyond molecular localization, enabling functional SRM that maps both the position and local environment of molecules in live cells which we will elaborate on in the next section.

## Advanced optical imaging methods for SMLM

SMLM has been extended to the imaging of additional dimensions of fluorescence signals besides the spatial information including spectra, fluorescence lifetime, polarization and orientation in addition to capturing the spatial information of individual molecules, which brings SRM into a functional imaging era.

### Spectrally resolved STORM (SR-STORM) and spectroscopic single-molecule localization microscopy (sSMLM)

In 2015, Xu's group introduced SR-STORM^78^ where fluorescence is split into two optical paths: one records the spatial PSF used for localization, while the other, after passing through a prism, records the corresponding emission spectrum. This dual detection allows each localization to be annotated with its spectral signature, enabling true-color super-resolution imaging far beyond the limits of conventional multicolor methods. In parallel, several groups including ours developed sSMLM^45,76,79,80^ using different dispersive elements such as a transmission grating to achieve similar capabilities. The primary advantage of SR-STORM/sSMLM is the ability to distinguish dyes with minimal spectral separations (<10 nm), which drastically reduces spectral crosstalk and permits high multiplexing (up to 4) in biological samples. Furthermore, spectral shifts provide a direct probe of the local nanoenvironment, including polarity changes, pH, or micro-heterogeneity around biomolecules. Applications range from multicolor labeling of subcellular structures and co-localization studies to high-throughput screening of probes with subtle spectral fingerprints.^24^

Fluorescence-lifetime single-molecule localization microscopy (FL-SMLM)^81^ combines the nanometer spatial resolution of localization microscopy with the temporal information of time-resolved fluorescence detection. In this approach, photon arrival times are measured either *via* time-correlated single-photon counting (TCSPC), and time-gated wide-field detection, or by employing fast single-photon avalanche diode (SPAD) array. This multidimensional readout enables lifetime-based probe discrimination, which is particularly powerful for fluorophores that are spectrally similar but differ in their decay dynamics. Beyond multiplexing, FL-SMLM allows nanoscale mapping of local environmental parameters such as refractive index, pH, and proximity to quenchers, and is uniquely suited for detecting Förster resonance energy transfer (FRET). Applications include resolving biomolecular conformations, mapping membrane heterogeneity, and probing dynamic nanoscale interactions in live cells. The principal advantage lies in the robustness of fluorescence lifetime as a contrast parameter instead of spectra- or intensity-based signals. It is largely insensitive to intensity fluctuations and spectral overlap, providing a reliable dimension for quantitative imaging. Fluorescence lifetime imaging microscopy (FLIM) has long been applied to polymer systems^82^ to probe regional polarity, viscosity, and phase heterogeneity, and FL-SMLM further extends this capability to the single-molecule level with nanometer resolution.

Polarization- and orientation-resolved SMLM (often termed SMOLM) provides access not only to the position of each emitter but also to the orientation and rotational mobility of its transition dipole. Early single-molecule polarization spectroscopy, exemplified by Prof. Weiss in the late 1990s, demonstrated that molecular orientation could be directly inferred from polarized emission.^83^ More recently, Lew's group integrated orientation detection into SMLM by engineering dipole-sensitive PSFs that encode orientation into distinct image features, allowing simultaneous retrieval of position and orientation.^84^ Other implementations use polarization beam splitters or excitation polarization modulation. The advantage of orientation-resolved imaging is its ability to reveal molecular order and an isotropy, detect conformational changes, and quantify rotational dynamics at nanometer resolution. This is particularly powerful when studying filamentous proteins, cytoskeletal organization, lipid membranes and amyloid fibrils, where alignment and mechanical constraints are key to function. In summary, advanced single-molecule imaging methods have greatly improved our understanding of functional information of the probing molecules and local environments besides the spatial information.

## Synthetic fluorophores for SMLM of fibers and polymers

Organic fluorescent dyes possess several advantageous photophysical properties in the context of SRM of polymers and fibers, including tunable emission spectra, higher photostability, greater photon yield, and facile synthetic modification and covalent integration during polymer synthesis or processing. In contrast, fluorescent proteins are relatively challenging to employ as they are designed to mature in biological systems. These characteristics make organic dyes far more suitable than fluorescent proteins for SRM of polymeric materials, where both photostability and precise molecular labeling are critical. Successful implementation of conventional and advanced SMLM methods in polymer imaging thus depends heavily on the selection of compatible fluorophores. Indeed, the development of switchable fluorophores and single-molecule imaging methodologies has become increasingly intertwined, with progress in one driving innovation in the other. In the context of polymer and fiber imaging, ideal probes also must be compatible with the non-aqueous imaging conditions that are atypical to biological SRM imaging. In this section, we will briefly introduce four commonly used organic fluorescent dyes in super-resolution imaging technology.

**Fig. 4:**
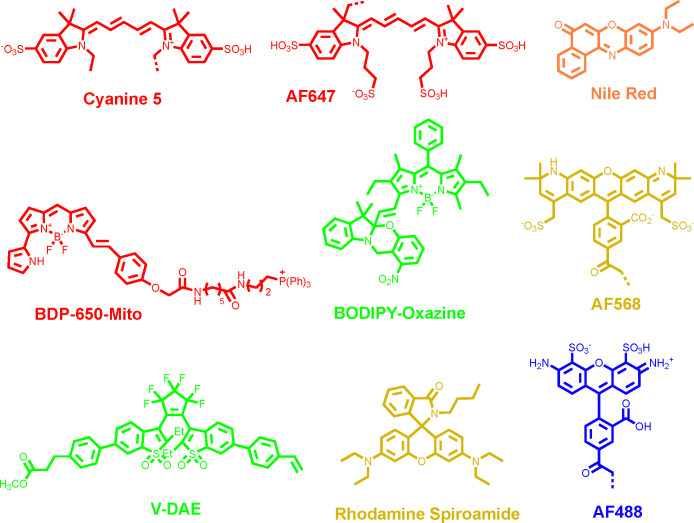
Chemical conformation of synthetic fluorescent dyes used in SRM for polymers and fibers.

Cyanine dyes represent a widely used class of fluorophores in SMLM because of their excellent brightness, high water solubility and facile conjugation to biomolecules.^85,86^ Recently, it has also been reported that the cyanine dye Alexa Fluor (AF) 647 can be effectively employed for polymer imaging, extending its use beyond biological systems.^87^ Cyanine dyes consist of two nitrogen-containing heterocycles one positively charged and the other neutral-linked by a polymethine chain (–CH groups with alternating single and double bonds) containing an odd number of carbon atoms (AF647 structure in Fig. 4).^88,89^ In contrast, modified cyanines or hemicyanines feature a polymethine bridge connected to nitrogen-containing functional groups on either side, forming a donor–π–acceptor system.^90–92^ The extended π-conjugation of cyanine dyes promotes efficient delocalization of photon energy, stabilizing electronic transitions and thereby shifting absorption into longer-wavelength regions. AF647 demonstrated improved photostability and a reduced tendency to cause self-quenching compared to Cy5.^93^ Cyanines typically undergo reversible photoswitching mechanisms (Fig. 3a).

Boron dipyrromethene (BODlPY/BDP) dyes are outstanding fluorophores characterized by high fluorescence quantum yields and brightness, making them excellent candidates for engineering SMLM probes. BODIPY fluorophores are composed of two pyrrole heterocycles bridged by a boron atom and a carbon framework (Fig. 4).^94–96^ Modification of functional groups around the BODlPY core can effectively tune their emission spectrum from 500 to 700 nm (Table 1).^97^ To extend their application in SMLM, BODlPY derivatives have been engineered with functional modifications to render them photoactivatable for PALM^40,98,99^ or capable of reversible binding for PAlNT imaging.^100^

**Table 1:** Photophysical properties of representative organic fluorescent dyes for SMLM

	*λ*_Abs_ a	*λ*_Em_ a	*Φ* b	*ε* c	*B* d	Non-aqueous^f^
AF488^102^	495	519	0.92	71	65	−
BDP-Oxazine	553	567	0.68	66	45	+
BDP650-Mito	646	660	0.52	102	53	−
Nile Red^e^	490/553	580/636	0.7/0.38	38/45	27/17	+
AF568^102^	578	603	0.69	91	63	−
AF647^102^	645	669	0.33	239	79	+
V-DAE	475	548	0.82	61	50	+
*RhB*	557	578	44.2	100	44	+

a Absorption (Abs) and emission (Em) maxima (nm).

b Fluorescence quantum yield.

c Extinction coefficients (cm^−1^ mM^−1^).

d Brightness *B* = *Φ* × *ε*.

e Nile Red dissolved in dioxane (top), methanol (bottom).

f Qualitative indication of whether the dye can work with polymer non-aqueous SMLM imaging conditions; solvents used are aqueous if not noted.

Nile Red is a widely used probe for lipid detection owing to its high affinity, specificity, and sensitivity to lipid hydrophobicity. Notably, its emission spectrum shifts from red in polar lipid environments to yellow in nonpolar lipids (Table 1). Nile red fluorophore could be used in PAINT by adding the functional anchor for the target. A zwitterionic short alkyl chain was introduced into Nile Red, which enabled reversible binding to the plasma membrane and built a PAINT image.^101^

Rhodamines represent a versatile class of inherently photoswitchable fluorophores that can be directly toggled using chemical reductants or engineered to generate photochromic and spontaneously blinking variants through spirocyclic ring opening.^103^ Rhodamine spiroamide (Fig. 4) molecules are known for their remarkable photochromic behavior undergoing UV-triggered ring-opening isomerization from a non-fluorescent to an emissive state. During the imprinting process, the resulting nanoscale deformations can locally bias the orientation and distribution of these fluorescent isomers, a feature that can be harnessed for PALM.^104^ This intrinsic flexibility, coupled with excellent photophysical properties including high brightness, good photostability, and efficient photoswitching has established rhodamines as one of the most widely used dye families in SMLM (Table 1). Many derivatives (Fig. 4), such as ATTO 488, AF488, and AF568, exhibit reversible and spontaneous fluorescence blinking by forming long-lived, non-emissive radical ion states, a process believed to arise from redox reactions or oxygenation of the triplet excited state. Consequently, additives such as thiols, ascorbate, and oxygen scavengers, along with deoxygenated imaging buffers, are routinely employed to regulate and optimize the blinking behavior of rhodamine-based fluorophores in SRM.^105^

Diarylethenes (DAEs) represent a versatile and rapidly expanding class of photochromic compounds that have attracted extensive attention over the past few decades (Fig. 4). Upon light irradiation, DAEs undergo a fast and reversible interconversion between their open- and closed-ring isomers, accompanied by a distinct color variation arising from modulation of the π-conjugation pathway. Remarkably, both the colorless open and the colored closed forms exhibit exceptional thermal stability, meaning that neither the cyclization nor the cycloreversion occurs spontaneously in the absence of light but instead requires illumination at specific wavelengths (Table 1). In addition, DAEs display outstanding fatigue resistance, maintaining their photochromic performance over numerous switching cycles in both solution and solid states. These unique features endow DAEs with exceptional potential for emerging applications in rapidly advancing fields such as SRM.^106^

## SMLM in polymer-based materials

### SMLM for visualizing the polymerization process

Building blocks sequences of synthetic polymers play an important role in the polymer macroscopic properties, such as their mechanical and thermal properties. Employing SRM to image polymeric processes will be advantageous for elucidating the structure–function relationship at the molecular level.

**Fig. 5:**
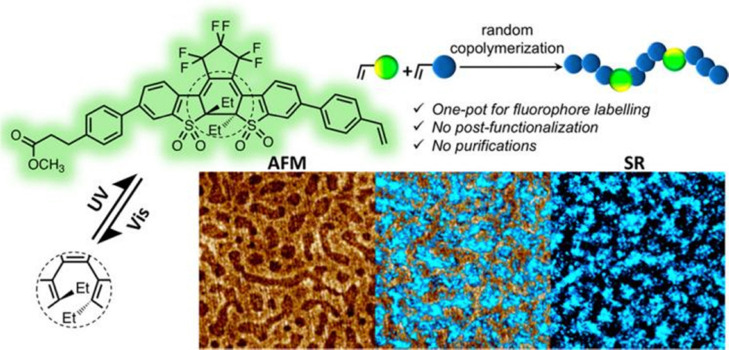
Visualizing synthesis process of polymers by SMLM. Left is an AFM phase image of thin films of PS/PMMA blends, right is a SRM image of thin films of PS/PMMA blends and they are partially overlaid in the middle. Scale bars are 1 µm. Reproduced with permission from ref. 107. Copyright 2018 American Chemical Society.

An early example of visualizing the synthesis process of polymers by SMLM was reported by Wang *et al.* in 2018.^107^ Wang developed a polymerizable photoswitchable fluorophore (V-DAE) (Fig. 5 top) that can be directly incorporated into polymer chains *via* RAFT copolymerization with styrene or methyl methacrylate (MMA), enabling precise control of labeling density without post-functionalization. Owing to its reversible photoisomerization between emissive and non-emissive states under UV (375 nm) and visible (473 nm) light, V-DAE allowed PALM imaging of polymer blends. Using V-DAE-labeled polystyrene (PS)/polymethyl methacrylate (PMMA) films, the authors visualized nanoscale phase-separated morphologies that correlated well with AFM data and further exploited the fluorophore's multiple switching cycles to obtain time-lapse images of dynamic structural evolution during solvent vapor annealing (Fig. 5). This work demonstrates a powerful strategy to integrate functional fluorophores through copolymerization and apply PALM to monitor polymer self-assembly and dynamics *in situ*.

In 2023, Chen's group developed coupled reaction approach toward super-resolution imaging (CREAST) for imaging single-catalyst polymerization at single-monomer resolution, in real time and at high reactant concentrations.^108^ Furthermore, the CREAST method is not limited to visualizing the ring-opening metathesis polymerization (ROMP) process, but can also be applied to monitor a broad range of chain-growth polymerizations, including living anionic, living cationic and living free radical polymerizations.

### SMLM for nanoscale morphology imaging of polymers

SMLM can not only visualize the polymerization process of polymeric materials but also their nanoscale morphology. An early demonstration of nanoscale morphology imaging of polymers using STORM was reported by Ross in 2014.^109^ In this study, STORM provided quantitative measurements of the sizes of demixed domains in a model PS/PMMA blend film that were in close agreement with those obtained by AFM.

**Fig. 6:**
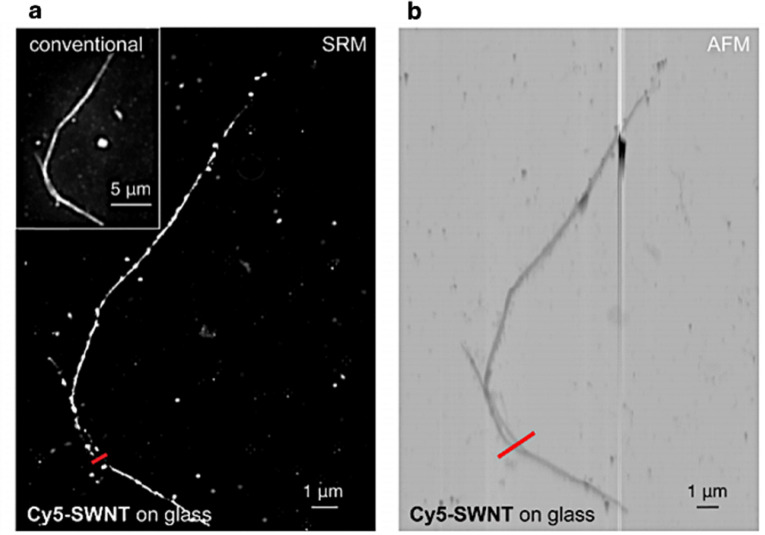
Conventional fluorescence, super-resolution and atomic force microscopy of dye-functionalized Cy5-SWNTs. (a) Super-resolution imaging, (b) AFM imaging. Reproduced with permission from ref. 110. Copyright 2018 American Chemical Society.

Xu's group demonstrated the power of SRM in materials science by directly visualizing cove-type graphene nanoribbons (cGNRs).^110^ In their study, ultra long cGNRs were synthesized *via* a bottom-up route and subsequently conjugated with Cy5 through copper-catalyzed click chemistry, enabling their detection on insulating substrates. While conventional fluorescence microscopy produced only blurred ribbon-like features (∼300 nm in width), SRM techniques based on fluctuation analysis (SOFl/SRRF) resolved individual nanoribbons with apparent widths of 40–50 nm and lengths up to 10 µm – the longest GNRs imaged to date. Notably, SRM distinguished isolated ribbons from entangled bundles and revealed sub-100 nm separations that remained inaccessible under diffraction-limited conditions. Comparison with AFM imaging of reference nanotubes validated these observations but also underscored the unique advantages of SRM as a non-destructive and high-throughput approach compatible with technologically relevant Si and Si/Si0_2_, substrates (Fig. 6). This case exemplifies how super-resolution methods, originally developed for biology, are now redefining the possibilities for structural characterization in nanomaterials.

### SMLM for imaging of the deformation of polymers

**Fig. 7:**
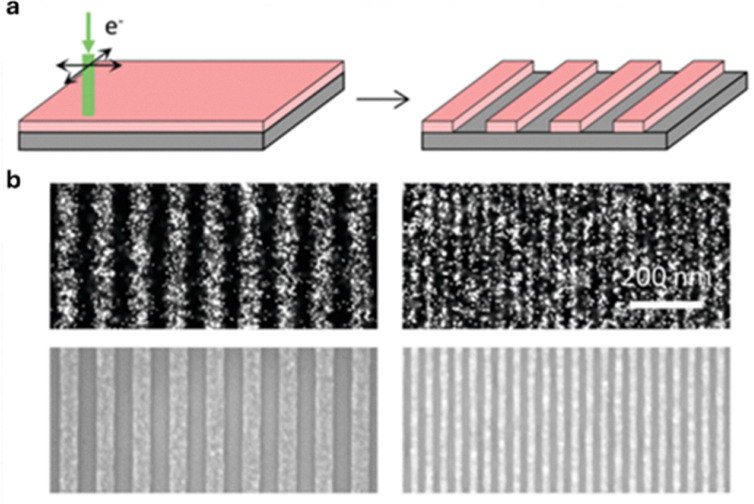
(a) Electron beam lithography is used to pattern 1 : 1 lines and spaces of 100 nm and 40 nm pitch in a 40 nm thick PMMA film. Mechanical deformation should be minimal in this process. (b) PALM imaging (upper) for mechanical deformation and comparison with scanning electron micrographs (lower). Reproduced with permission from ref. 111. Copyright 2019 Royal Society of Chemistry.

SMLM can also visualize the nanoscale deformation process of polymers. In 2019, Wang employed PALM to investigate nanoscale mechanical deformation in polymer films patterned by thermal nanoimprint lithography.^111^ PMMA films doped with a photoactivatable rhodamine spiroamide fluorophore enabled PALM imaging, allowing simultaneous determination of fluorophore positions and orientations through point-spread function fitting. The reconstructed orientation maps revealed anisotropic alignment of fluorophores in regions as small as 20 nm, clearly distinguishing deformed areas from neighboring undisturbed domains. Control experiments using electron-beam lithography confirmed that the observed orientation changes arose from mechanical strain rather than imaging artifacts. The agreement between PALM-based maps and AFM data further validated the method (Fig. 7). Overall, PALM was essential for overcoming the diffraction limit and directly visualizing nanoscale deformation, providing strong support for the conclusion that super-resolution orientation microscopy can serve as a powerful tool for probing nanomechanical processes in polymers.

As a powerful tool, SMLM has applications in polymer-based materials that extend beyond the aspects mentioned above, including phase separation,^112^ interfacial heterogeneity,^113^ and nanoscale distribution imaging.^114^

### SR-STORM for imaging of the constituents of the polymer

**Fig. 8:**
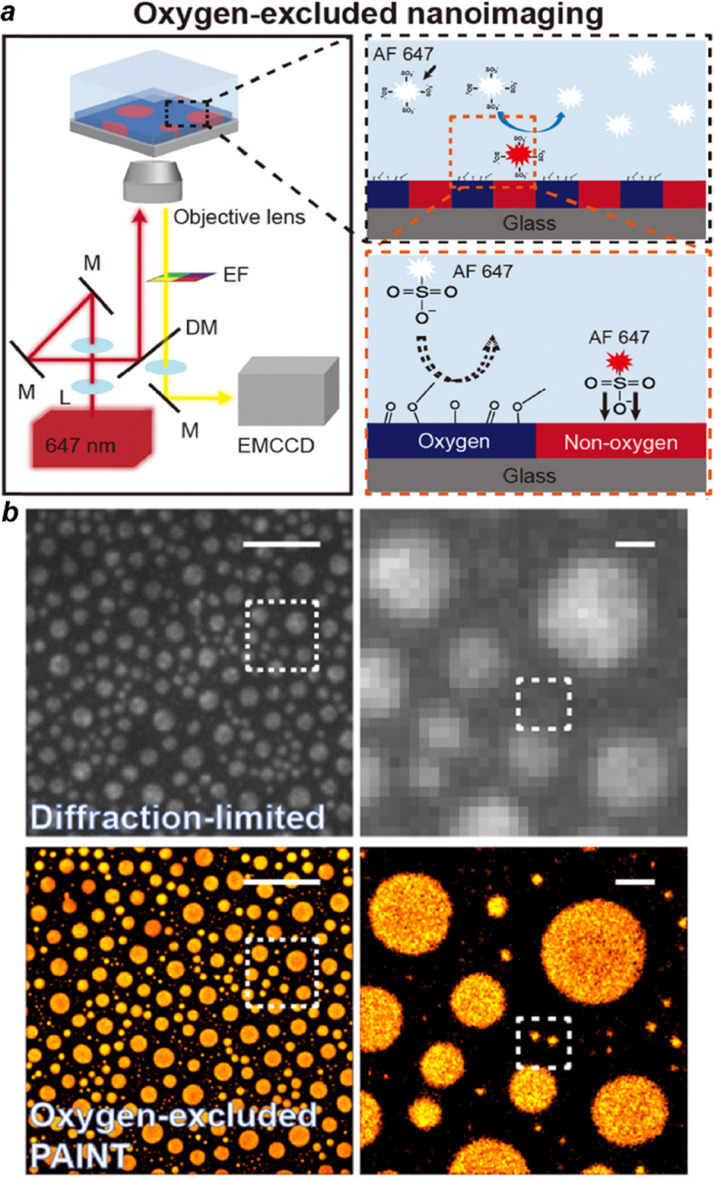
(a) Scheme of oxygen-excluded nanoimaging of a polymer blend film. (b) Representative super-resolution images of the non-oxygen region in the phase-separated PS-PMMA polymer blend film using Alexa Fluor 647 dyes. Scale bars are 5 µm Reproduced with permission from ref. 112. Copyright 2025 American Chemical Society.

Recently, Kim's group employed an oxygen-excluded super-resolution imaging strategy based on PAlNT to selectively visualize non-oxygen domains in polymer blend films.^112^ AF647 was introduced as the probe, where its strong electrostatic repulsion with oxygen atoms in polymer side chains prevented binding to oxygen-rich regions while enabling transient interactions with oxygen-free domains. This selective labeling provided nanoscale contrast without additional sample modification. STORM imaging of the binding events achieved spatial resolutions down to ∼15–20 nm, enabling direct visualization of nanoscale phase separation, domain identification, and hierarchical structural features in PS/PMMA and other polymer blends (Fig. 8).

Park's group integrated SR-STORM for embedded probes and spectrally resolved PAINT for surface binding, they distinguished internal *versus* interfacial polarity, differentiated side-chain functionalities, and visualized phase separation in blends at ∼30 nm resolution.^77^ This non-destructive optical approach thus goes beyond AFM or contact-angle analysis by adding chemical specificity, establishing SMLM as a powerful tool for probing nanoscale heterogeneity in multi-component polymers.

Wöll's group developed a FL-SMLM to quantify local water content in polymer gels.^78^ This approach exploits the efficient fluorescence quenching of red-emitting dyes by H_2_O, in contrast to the negligible effect of D_2_O, such that systematic lifetime measurements in H_2_O/D_2_O mixtures provide a direct readout of local hydration. By covalently incorporating the dye into the polymer network, this technique enables nanoscopic mapping of water distribution in both swollen and collapsed gel states.

### SMLM for DNA origami imaging

**Fig. 9:**
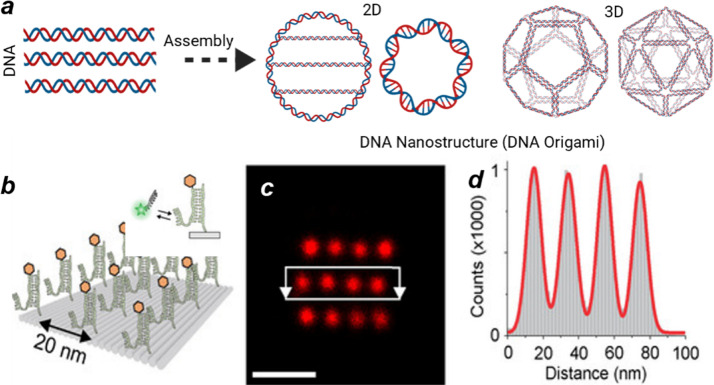
(a) Illustration of programmable DNA materials to assemble DNA origami; (b) a schematic shows DNA-PAINT imaging of a DNA origami sample designed as a reference target for SRM imaging quality evaluation; (c) the corresponding DNA-PAINT image and (d) the histogram of relative localizations distance in c. Scale bars is 50 nm. Reproduced with permission from ref. 115. Copyright 2021 European Chemical Societies Publishing.

Programmable DNA materials have enabled transformative applications in biosensing,^116^ precision medicine,^117^ and DNA-based computing.^118^ As a polymer of nucleotide monomers, DNA possesses well-defined Watson–Crick base-pairing rules that provide sequence-specific binding with predictable thermodynamics and kinetics.^119^ Building on the pioneering work of Seeman and Rothemund^120,121^ these principles have enabled the design of sophisticated 2D and 3D DNA origami structures that organize biomolecules with nanometer precision (Fig. 9a). These structures are assembled by folding a long single-stranded DNA scaffold into defined shapes using hundreds of short “staple” strands through complementary base pairing, creating addressable nanoscale architectures. DNA origami serves as an exceptional platform for validating SRM,^75^ providing precisely engineered nanostructures (*e.g.*, DNA nanogrid and nanoruler) with programmable binding sites at known positions (Fig. 9b and c).^115^

The integration of DNA origami for structural fabrication with DNA-PAINT for high-resolution visualization exemplifies how DNA's molecular programmability supports both the construction and interrogation of nanoscale architectures.^122^ DNA-PAINT leverages this programmability by incorporating “docking” strands at specific sites on the origami, which transiently bind fluorescent “imager” strands in solution (Fig. 3d). The resulting stochastic on–off fluorescence enables single-molecule localization with sub-5 nm precision (Fig. 9d), while continuous imager exchange eliminates photobleaching and supports long-term, quantitative, and multiplexed 3D imaging. Beyond serving as biological model systems, DNA-based assemblies are being increasingly viewed as a blueprint for designing programmable synthetic polymers materials that combine molecular precision, structural tunability, and optical addressability to enable the next generation of functional nanoscale imaging and fabrication.

### SMLM for DNA hierarchical organization in natural systems

**Fig. 10:**
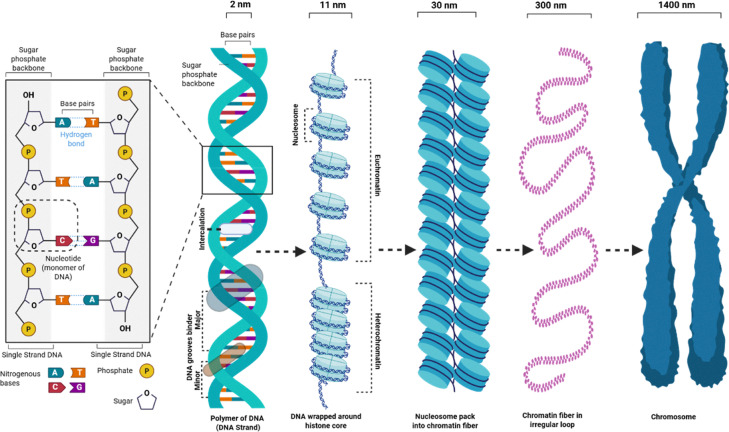
Illustration of the hierarchical structure of the DNA polymer, chromatin fibers and higher-order assemblies.

**Fig. 11:**
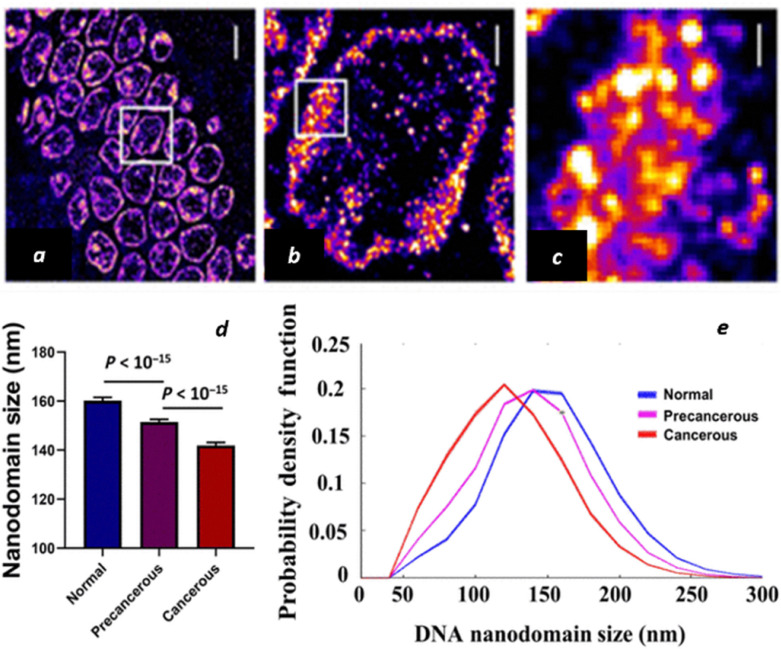
Visualization of DNA polymer organization using Hoechst-Cy5. (a) STORM image of genomic DNA revealing heterogeneous chromatin nanodomains. (b) Magnified view showing detailed DNA polymer organization. (c) High-magnification view demonstrating nanometer-scale DNA compaction patterns. Scale bars: 5 µm (a), 1 µm (b), 200 nm (c). Reproduced with permission from ref. 123. Copyright 2022 American Association for the Advancement of Science.

The polymeric nature of DNA underpins its dual role as a genetic information carrier and as a dynamic template for replication and repair, which are processes essential for cellular viability and organismal survival. In biological systems, DNA exhibits a hierarchical organization spanning multiple length scales: from nucleosomes (∼150 base pairs, ∼10 nm) to 30-nm chromatin fibers (several kilobase pairs), chromatin compartments hundreds of nanometers in size (megabase pairs), and chromosomes extending up to several micrometers (hundreds of megabase pairs) (Fig. 10). SMLM has made it possible to visualize these higher-order DNA and chromatin architectures with nanometer resolution. Liu and co-workers developed Hoechst–Cy5, a covalently linked fluorescent conjugate that combines Cy5's superior photoswitching properties with Hoechst's sequence-specific DNA binding, enabling molecular-scale imaging of genomic DNA organization.^123^ Using this probe, they revealed heterogeneous chromatin nanodomains that were previously undetectable by conventional microscopy (Fig. 11).

Functional SMLM imaging further captured dynamic DNA compaction states, revealing progressive nanodomain shrinkage from normal to precancerous to cancerous tissue, reflecting the disruption of higher-order chromatin folding during tumorigenesis. Two-color SMLM additionally mapped DNA polymers relative to nuclear machinery, showing how open chromatin conformations facilitate RNA polymerase II access, while fragmented nanodomains associate with the nuclear lamina during cancer progression. This approach successfully distinguished DNA architectures across diverse cell types, from open chromatin in intestinal stem cells to condensed configurations in quiescent immune cells, providing molecular-level insights into genome organization and transcriptional regulation. Importantly, analyses of tissues from Lynch syndrome patients revealed chromatin abnormalities even in histologically normal samples, suggesting that nanoscale chromatin signatures may serve as early biomarkers of genetic predisposition. In short, this work establishes a paradigm for functional DNA polymer imaging, which not only advances our understanding of genome organization in health and disease but also provides conceptual and methodological inspiration for imaging and interpreting structure–function relationships in synthetic polymer systems, where hierarchical organization and dynamic conformational changes similarly govern material properties.

### Phalloidin-PAlNT for actin fiber

**Fig. 12:**
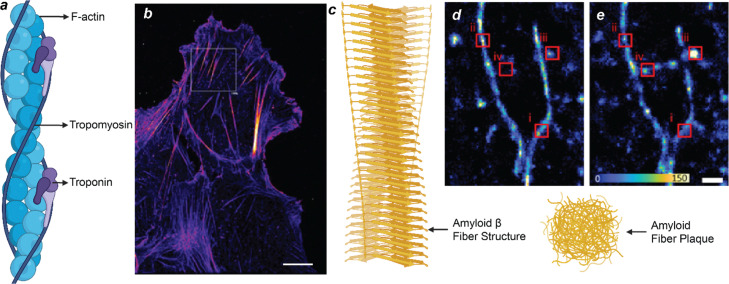
(a) and (b) Illustration (a) of the structure of Actin polymer and (b) a representative PAINT image of actin filaments of a U2OS cell.^124^ (c)–(e) illustration of Amyloid fibers and higher-order assemblies and SMOLM images (d) and (e) to quantify amyloid fiber stability and remodeling of Aβ42 fiber (d) before and (e) after 10 min of ThT-induced remodeling; Scale bars: (b) 10 µm, (d) and (e) 300 nm. Panels (b) and (d) and (e): Reproduced with permission from ref. 124 and 115. Copyright 2024 ScienceDirect and 2021 European Chemical Societies Publishing, respectively.

Actin filaments derive from the ATP-regulated polymerization of globular actin into dynamic, semiflexible helices that support cellular mechanics, membrane remodeling, and force transmission. Despite their divergent biological roles-cytoskeletal function. Understanding the nanoscale organization of actin filaments in cells and intact tissues remains challenging due to the loss of phalloidin signals during expansion and the limited optical accessibility of thick samples. To address the long-standing challenge of quantitatively super-resolving F-actin in mechanically fragile membrane protrusions, where conventional phalloidin-based dSTORM suffers from labeling loss and biased quantification. Hu's group demonstrated a phalloidin-PAlNT, exploiting the intrinsic and chaotrope-enhanced dissociation of dye-conjugated phalloidin. By adding moderate concentrations of KSCN (potassium thiocyanate), phalloidin-F-actin dissociation and sampling rates are increased without denaturing cellular structures, yielding consistent nanoscale reconstructions across the entire cell. Using U2OS cells and both immortalized and primary dendritic cells, phalloidin-PAlNT reveals thin actin filaments, preserves delicate membrane protrusions, and enables unbiased quantification of local actin densities (Fig. 12b).^124^ More importantly, the method captures F-actin redistribution from podosomes to cytoskeletal filaments upon lipopolysaccharide stimulation, demonstrating its utility for probing stimulus-induced cytoskeletal remodelling at the nanoscale.

### SMOLM for amyloid fiber imaging

Amyloid fibers emerge from the misfolding and hierarchical stacking of β-sheet-rich peptide segments, producing highly ordered, rigid fibrils with exceptional mechanical and chemical stability. Despite their divergent biological roles – cytoskeletal function *versus* pathogenic aggregation – both systems demonstrate how subtle variations in conformation, intermolecular bonding, and packing geometry direct the emergence of supramolecular architectures. A major challenge in understanding amyloid-related pathogenesis is the inability to resolve how individual fibrils grow, reorganize, and undergo structural decay at the nanoscale. Lew's group develops single-molecule orientation localization microscopy (SMOLM), a polarization-based method that simultaneously reports nanometric emitter locations and full three-dimensional dipole orientations with 18-nm spatial resolution (Fig. 12d and e).^125^ Using orientation distributions as a structural reporter, the authors reconstruct the nano-architecture of amyloid assemblies undergoing elongation, heterogeneous bundling, and thermal degradation. SMOLM reveals that fibril growth proceeds through non-colinear addition of protofilaments, leading to distinct orientation domains, while decay is marked by progressive disorder and loss of β-sheet alignment (Fig. 12c). Importantly, the method discriminates single filaments from bundled structures resolves twisting and local curvature, and captures dynamic transitions in individual fibrils. Together, these results demonstrate that SMOLM provides a uniquely sensitive view of amyloid morphology beyond localization-only imaging, enabling direct visualization of growth pathways and failure modes. The work establishes orientation-resolved single-molecule microscopy as a powerful tool for dissecting amyloid assembly mechanisms and for probing nanoscale structural heterogeneity relevant to neurodegenerative disease.

## Conclusions and perspectives

SRM technologies, with their exceptional spatial resolution, single-molecule precision in imaging and quantification, multiplexing capability, and relatively low invasiveness, have rapidly emerged as indispensable tools across the life and physical sciences. Over the past two decades, these techniques have evolved far beyond their initial applications in cell biology and are now driving discoveries in a wide spectrum of fields, including neuroscience,^126,127^ immunology,^128,129^ structural biology,^130,131^ drug discovery^75,132^ and nanomaterials science.^59,133^ In this Focus article, we introduced the imaging principles of SMLM, utilized organic fluorophores and then discussed the application of SMLM in polymer-based materials.

Despite its transformative impact, SMLM remains constrained by several technical and practical challenges. Limitations in detector performance such as quantum efficiency, temporal resolution which continue to affect imaging speed and precision. Likewise, the absence of streamlined and accessible data-processing pipelines has become a major bottleneck as datasets grow in both scale and complexity. From an application standpoint, the current palette of fluorophores still falls short in simultaneously achieving brightness, photostability, and spectral separability, restricting multiplexed and long-term imaging. Finally, the technical expertise required for both experimental operation and data interpretation limits broader adoption beyond specialized laboratories.

As SMLM continues to penetrate polymer materials research, an increasingly important consideration concerns the extent to which covalent incorporation of fluorophores perturbs the intrinsic structure and dynamic behavior of the host polymer. Unlike biological macromolecules, synthetic polymers encompass broad chemical diversity and exhibit environment-dependent chain mobility, packing motifs, and phase behavior that may be highly sensitive to the steric volume, charge distribution, or local polarity introduced by labeling groups. Indeed, recent demonstrations using photochromic and spontaneously blinking dyes within polymer matrices highlight that even low fractional incorporation can modulate chain relaxation, microphase organization, or stress-induced reconfiguration. These concerns underscore the need for systematic strategies for probe selection, including minimizing label density, employing chemically orthogonal anchoring sites, and introducing flexible linkers to spatially decouple the dye from the backbone. Parallel development of non-perturbative modalities, such as PAINT-like transient binders, environment-sensitive probes, or end-group-specific tagging will be essential for ensuring that SMLM reports authentic structural and functional features rather than label-induced artifacts. Establishing standardized validation metrics that correlate photophysics, chain conformation, and mesoscale morphology will further enhance the reliability of SMLM as a quantitative tool for polymer science.

Parallel to advances in fluorophore chemistry, the rapid evolution of expansion microscopy (ExM) offers a compelling opportunity to synergistically extend the reach of SMLM into previously inaccessible polymer architectures.^134–138^ While ExM has transformed biological imaging through physical magnification, the translation of hydrogel embedding, homogenization, and isotropic swelling to synthetic polymers is non-trivial. Many polymers lack intrinsic anchoring sites, exhibit solvent-dependent swelling behaviors, or undergo morphological rearrangement during gelation-factors that may compromise structural fidelity. Nonetheless, emerging ExM variants that use carbodiimide-mediated anchoring, click-based grafting, or supramolecular retention chemists suggest feasible pathways for adapting expansion to block copolymers, nanofibers, supramolecular assemblies, and porous or heterogeneous polymer networks. When coupled with multidimensional SMLM-including spectral, lifetime, and orientation modalities ExM could enable molecular resolved mapping of phase-separated domains, confined chain dynamics, and local heterogeneity across micrometer-to-millimeter fields of view. Critical directions include defining polymer-specific anchoring strategies, quantifying expansion isotropic relative to native mechanical moduli, and designing fluorophores capable of retaining their photoswitching behavior throughout the swelling workflow. Based on these developments promise to establish SMLM-ExM integration as a powerful multi modal framework for interrogating polymer materials across fundamentally new length scales.

Future advances in SMLM are expected to address these challenges through synergistic progress in hardware, chemistry, and computation. On the instrumentation side, next-generation detectors with enhanced sensitivity, low noise, and high acquisition rates will enable faster and more reliable imaging even under demanding experimental conditions. Parallel advances in fluorophore chemistry to yield brighter, more photostable, and environmentally responsive dyes will extend the reach of multiplexed, long-term, and *in vivo* studies. Equally transformative is the integration of artificial intelligence (AI) into image acquisition and analysis. Deep learning-based frameworks such as ANNA-PALM^139^ and DI-STORM^140^ have demonstrated the ability to reconstruct super-resolution images from reduced datasets, while methods like BGnet^141^ and SpecUNet^142^ enable rapid background correction, denoising, and spectral quantification. These AI-driven tools not only accelerate image reconstruction but also expand the analytical frontier toward automated clustering, spatial statistics, and dynamic modeling of molecular assemblies. When combined with complementary approaches such as electron or atomic force microscopy, SRM will increasingly offer a multi-scale and multimodal understanding of complex systems.

### Bridging biological and artificial fiber systems

Looking ahead, SRM provides a powerful common ground for studying biological and artificial fibrous systems—two domains that share fundamental questions about structure, dynamics, and function but have evolved largely in parallel. In biological systems, SRM reveals how protein and chromatin fibers assemble, reorganize, and regulate cellular processes through dynamic phase transitions. In artificial systems, it uncovers how polymer chains and nanofibers self-assemble, crystallize, or undergo stress-induced reconfiguration. Integrating insights from both domains, especially through AI-enabled analysis, could lead to a unified framework for understanding how molecular organization encodes function across living and synthetic materials. For instance, concepts such as viscoelastic relaxation, cooperative mobility, and defect healing that are central to polymer science may inform models of chromatin and cytoskeletal organization, while mechanisms of active remodeling in cells may inspire new strategies for designing adaptive, self-healing, or stimuli-responsive materials.

## Author contributions

Conceptualization: S. R., X. G, and Y. Z.; investigation, visualization and writing – original draft: S. R., X. G., M. A. S., Y. L., H. M.; writing – review & editing: Y. Z.; funding acquisition and supervision: Y. Z.

## Conflicts of interest

There are no conflicts to declare.

## Data Availability

No primary research results, software or code have been included and no new data were generated or analysed as part of this review.
